# Bibliographical—Useful Hints for the Busy Dentist

**Published:** 1893-04

**Authors:** 


### EDITORIAL.

### Bibliographical—Useful Hints for the Busy Dentist.

This volume, consisting of five hundred and sixty-seven useful hints for the busy dentist, by Wm. H. Steele, D.D.S.; published by the Wilmington Dental Manufacturing Co., Philadelphia, came to hand recently.

The aim of the work will be readily understood by the following from the preface: "My intention has been to furnish an answer as accurately and with sufficient detail in as compact and accessible a form as possible, to all the questions and difficulties that may arise in our every day life at the chair or bench."

This is a volume of hints and suggestions, in the main, gathered from our periodical literature; gathered from the best writers in the profession. These hints will be of very great interest to the young practitioner especially, and even to the older whose time may be so closely occupied in practice as to prevent a reading of our periodical literature. We may say, however, that if such a work as this is made wholly to take the place of the reading of our periodical literature it might thus stand in the way of the best interests of the science of our profession. In one aspect this is an age of paragraphing, and in some departments, at least, this method of communicating occupies more than its proper share of space and attention. The intelligent dentist, however, will understand how to maintain a proper balance in this respect.

We can heartily recommend this work to the profession.

